# Over-Expression of Telomere Binding Factors (TRF1 & TRF2) in Renal Cell Carcinoma and Their Inhibition by Using SiRNA Induce Apoptosis, Reduce Cell Proliferation and Migration Invitro

**DOI:** 10.1371/journal.pone.0115651

**Published:** 2015-03-02

**Authors:** Deeksha Pal, Ujjawal Sharma, Shrawan Kumar Singh, Nandita Kakkar, Rajendra Prasad

**Affiliations:** 1 Department of Biochemistry, PGIMER, Chandigarh, India; 2 Department of Urology, PGIMER, Chandigarh, India; 3 Department of Histopathology, PGIMER, Chandigarh, India; UCSF / VA Medical Center, UNITED STATES

## Abstract

Telomere binding factors viz. TRF1 and TRF2 are a part of sheltrin complex that are present exclusively at the ends of chromosomes. These factors play an important role in maintaining chromosomal integrity at the ends. However, their status and role are not clear in renal cell carcinoma (RCC). Therefore, the present study was conducted to evaluate TRF1 and TRF2 expressions in RCC tissues. Further, the role of these factors involved in tumorigenesis was elucidated by gene silencing using siRNA in RCC cell line (A498). The present study documented a significant over-expression of TRF1 (P = 0.005) and TRF2 (P = 0.0048) mRNAs by real time PCR in RCC tissues as compared with adjacent normal kidney tissues. Immunohistochemistry studies also revealed higher expression of TRF1 and TRF2 proteins in RCC. Moreover, TRF1 or TRF2 gene silencing using siRNA showed marked reduction in proliferation of RCC cells (P = 0.000). Further, significantly induced cell cycle arrest (P = 0.000) and apoptosis of RCC cells (P = 0.000) was documented upon TRF1 or TRF2 gene silencing. Henceforth, the results deduce that TRF1 or TRF2 inhibitions play an important role in the induction of apoptosis in A498 cells, which may serve as a potential therapeutic target in RCC.

## Introduction

Renal cell carcinoma (RCC) is the most common renal tumor attributing approximately 90% of all renal malignancies [1]. It is a constellation of malignancies of different histological subtypes arising from the renal parenchyma [2]. RCC comprises about 80% of conventional clear cell type, 15% papillary type and 5% of other types [3]. RCC does not respond to radiotherapy, hormonal therapy and chemotherapy [4]. It does not manifest any early symptoms. Early diagnosis is very difficult in RCC; however, in most of the cases it is detected incidentally on radiological examination.

The telomeric repeat binding factors TRF1 and TRF2 play an important role in T-loop structure by directly binding with the double stranded region of the telomere [5, 6]. Telomeres are DNA-protein structures that protect chromosomes ends from degradation and fusion [7]. Telomeres are composed of repetitive DNA sequences of TTAGGG repeats and telomere binding proteins [8]. The electron microscopic studies based model revealed that “T-loop” and displacement (D) loop are formed by the invasion of 3ʹ overhang region to the double stranded telomeric region. TRF1 has the ability to induce bending, looping and pairing of duplex telomeric DNA activities that could facilitate the folding back of the telomere [9]. TRF2 play a role in the invasion of 3ʹ single stranded TTAGGG repeat tail into duplex telomeric DNA. Thus, telomere length regulation by T-loop along with TRF1 and TRF2 proteins are required to maintain the telomeres length homeostasis [10].

Several studies have documented that the upregulation of TRF1 and TRF2 have been associated with lung cancer [11] and gastric cancer [12]. Conversely, these genes were found to be down regulated in malignant hematopoietic cells [13] and breast cancer [14]. However, the expression of telomeric binding proteins (TRF1 and TRF2) and their role in the tumorigenesis of RCC are still unknown. In this study, TRF1 and TRF2 expressions at transcriptional and translational level were appraised in RCC. Subsequently, TRF1 and TRF2 inhibitions by their gene silencing may limit the proliferative potential as well as induce apoptosis and cell cycle arrest in RCC cell line. These findings raise the intriguing possibility that TRF1, TRF2 inhibition can be used as a therapeutic approach by directly targeting telomere integrity.

## Materials and Methods

### Patients

The present study was approved by the institute ethics committee and informed consent was obtained from patients. Following nephrectomy, tissue samples were taken from the tumor and grossly normal renal parenchyma separately. The samples were snap frozen in liquid nitrogen and stored at -80°C till further use. Tumor staging was done according to TNM staging [15] and grading of clear cell RCC type was performed by Fuhrman grading [16]. Detailed clinical characteristics of patients are given in Table 1.

**Table 1:** Clinical characteristics of patients.

Patients (n)	92
Gender	
Male	64 (69.6)
Female	28 (30.4)
Age (years), mean±S.D.	53±13.8
BMI, mean±S.D.	23.67±0.164
Commonest presenting complaints n (%)	
Hematuria	47 (51.0)
Flank pain	29 (31.5)
Both	20 (21.7)
High fever	22 (23.9)
Incidental radiological examination	15 (16.3)
Histologic subtypes, n (%)	
Clear cell	66 (71.7)
Papillary	12 (13.1)
Sarcomatoid	8 (8.7)
Oncocytoma	6 (6.5)
TNM Stage, n (%)	
T1N0M0	35 (38.0)
T2N0M0	18 (19.6)
T1N1M0	3 (3.3)
T2N1M0	3 (3.3)
T3N0M0	28 (30.4)
T3N1M0	1 (1.1)
T4N0M0	4 (4.3)
Types (Papillary), n (%)	
I	8 (66.6)
II	4 (33.3)
Fuhrman Grade (Clear cell), n (%)	
I	14 (21.2)
II	33 (50.0)
III	12 (18.2)
IV	7 (10.6)

S.D. Standard deviation

### Real-time PCR

Total RNA was isolated from normal and tumor tissue using PureLink RNA mini kit (Invitrogen) as per manufacturer’s instruction. Reverse transcription of 1μg of RNA was performed with first-strand c-DNA synthesis using SuperScript^III^ kit (Invitrogen, CA, USA). Real-time analysis was performed on 7300 RT-PCR system (Roche Indianapolis, IN) using the light cycler RNA Master SYBR Green kit (Roche Diagnostics, Indianapolis). The oligonucleotide sequences of the primers were as follows:

**Table:** 

TRF1	Forward 5ʹ-CCACATGATGGAGAAAATTA AGAGTTAT-3ʹ
	Reverse 5ʹ-TGCCGCTGCCTTCATTAGA-3ʹ
TRF2	Forward 5ʹ-ACCAGGGCCTGTGGAAAAG-3ʹ
	Reverse 5ʹ-GGTGGTTGGAGGATTCCGTA-3ʹ
β-actin	Forward 5ʹ-CGAGCGCGGCTACAGCTT-3ʹ
	Reverse 5ʹ-CCTTAATGTCACGCACGATTT-3ʹ

TRF1 and TRF2 expression levels were normalized to β-actin for each tumor sample and calculated relative to normal renal tissue (control) using the following equation [17].

$$\text{Fold change }={\text{ 2}}^{-\left(\text{Tumor }△\text{Ct }\_\text{ Control }△\text{Ct}\right),}\text{where }△\text{Ct }=\text{ Ct }\left(\text{TRF1 or TRF2}\right)-\text{ Ct }\left(\beta -\text{actin}\right).$$

### Immnunohistochemical staining for TRF1 and TRF2

Goat polyclonal IgG antibody directed against the human TRF1 (sc-1977, Santa Cruz, USA) or TRF2 (sc-9143, Santa Cruz, USA) was used to detect TRF1 and TRF2 in all the samples (92) of RCC, according to the method as already described in detail previously [18]. Briefly, 4 mm tissue sections were cut, dewaxed, and incubated in absolute methanol solution with 0.3 mL of hydrogen peroxide for 30 min. Antigen retrieval was carried out by boiling the slides in 10 mM citrate buffer, pH 6.0 for 15 min. Slides were then treated with blocking serum for 10 min, after which they were incubated with 1:200 diluted primary antibody (anti TRF1/TRF2) at 37° C for 60 min followed by 1:50 diluted HRP anti-goat IgG (Bangalore Geni, India). Chromogen detection was performed with diaminobenzidine (DAKO Corp., Carpinteria, CA) solution (0.5 mL of stock diaminobenzidine in 4.5 mL of Tris buffer with 20 mL of hydrogen peroxide). Slides were counterstained with hematoxylin and photographed.

### Cell culture

Human renal carcinoma cell line A498was derived from National Centre for Cell Sciences, Pune, India, and maintained in Dulbecco’s modified eagle’s medium supplemented with 10% heat-inactivated fetal bovine serum, 100 U/mL penicillin and 100 μg/mL streptomycin. The cells were allowed to 60–80% confluent at 37°C in a 5% CO_2_ atmosphere.

### siRNA treatment

Cells were treated with 100μM of each scrambled siRNA (SIC001, Mission siRNAs, sigma aldrich) and siRNA specifically targeting TRF1 (EHU0114821, Mission siRNAs, sigma aldrich) or TRF2 (EHU042991, Mission siRNAs, sigma aldrich) alongwith lipofectamine for 6 hr. Then 2X normal growth medium was added. After 24 hr 2X media was replaced with 1X growth medium to reduce toxicity.

### MTT assay

Briefly, 5000 exponentially growing cells per well were seeded in 96-well plates. After siRNA treatment and 4 hr prior to completion of incubation period, 10 μl of 3-[4, 5-dimethylthiazol-2-yl]-2, 5 diphenyl tetrazolium bromide (MTT; Sigma-Aldrich) reagent was added to each well. After 4 hr, MTT solution was removed and the blue crystalline precipitate in each well was dissolved in Dimethyl sulfoxide. Cells were quantified at 570 nm using a microplate reader (uQuant, BioTek Instruments).

### Wound healing assay

Cells were allowed to grow in 6 well plates. Cells were treated with respective siRNAs and after 24 hr; a wound was generated by scratching with a pipette tip at the base of a six well plate. Photographic images were taken from each well at 0 hr and 48 hr after creation of the wound. The distance that cells migrated through the area created by scratching was determined by measuring the wound width at the above times and subtracting it from the wound width at the start.

### Cell cycle analysis using flow cytometry

After 96 hr of siRNA treatment, Cells were harvested and centrifuged at 2000 rpm for 5 min. Cells were re-suspended in the 300 μl PBS and 2.5 μL RNase A (20 mg/mL) and 2 μL of PI (5 mg/mL) were added to the re-suspended cells. Then, the cells were analyzed for cell cycle analysis by flow cytometery (BD FACSCalibur).

### Detection of apoptosis by flow cytometry

Apoptosis was detected by flow cytometric analysis of Annexin V–FITC versus PI assay (Vibrant apoptosis assay, V-13242, Molecular Probes, Eugene, OR, USA). Briefly, adherent cells were harvested after 96 hr of siRNA treatment and suspended in the annexin-binding buffer (1x10^6^ cells/mL). Thereafter, cells were incubated with annexin V–FITC and PI for 15 min at room temperature in the dark and immediately analyzed by flow-cytometry (BD FACSAria). The data are presented as a bi-parametric dot plots, annexin V–FITC versus PI.

### H & E staining

After the 96 hr of siRNA treatment, cells were harvested and collected on a poly-L-lysin coated slide by centrifugation in a cytospin centrifuge (Sigma) at 1000 rpm for 10 min. Cells were stained with heamatoxylin for 1 min and then counterstained with eosin for 30 sec. Cells were mounted with DPX and visualized under microscope and photographed (Olympus).

### Statistical analysis

Statistical analysis was performed with SPSS program (version 20.0; SPSS Inc., Chicago, IL). The difference of gene expression in normal and tumor renal tissues was assessed by one sample t- test. Comparisons of mean values were performed using ANOVA. A P-value of <0.05 was considered significant.

## Results

### Upregulation of TRF1 and TRF2 genes and proteins in RCC

**Fig 1:**
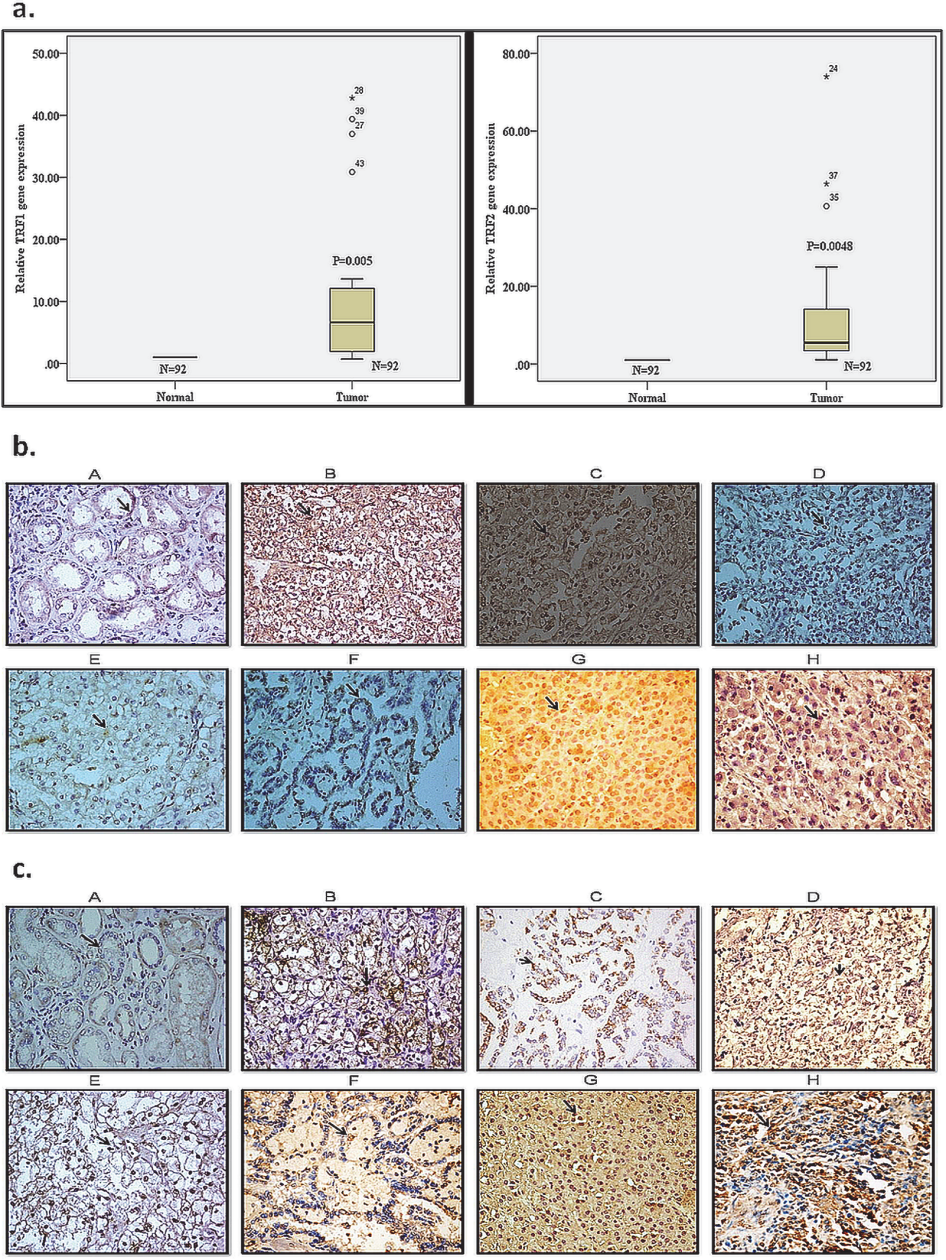
mRNA and protein expression of TRF1 and TRF2 (a). The result are presented as box plot showing TRF1 and TRF2 mRNA expression level (A) TRF1 (B) TRF2 in normal and RCC tissues were determined by quantitative real time PCR. The bottom and top edges of the box located at the 25^th^ and 75^th^ percentiles respectively of the sample. The centre horizontal lines are drawn at the median of the sample. β-actin mRNA levels were used to normalize TRF1 and TRF2 gene expression. Statistical analysis was done by one sample t- test. P<0.05 was considered significant. **(b) & (c). Immunohistochemical staining of TRF1 and TRF2 protein respectively.** IHC was performed for TRF1 in all the cases (92) of RCC. The representative figures of (A) Normal renal parenchyma; (B) grade I clear cell RCC; (C) grade II clear cell RCC; (D) grade III clear cell RCC; (E) grade IV clear cell RCC; (F) papillary RCC; (G) oncocytoma; (H) sarcomatoid were shown (x40).

We observed a significantly higher level of TRF1 (P = 0.005) and TRF2 (P = 0.0048) gene expression in RCC tissue as compared with normal renal parenchyma (Fig. 1A). However, no correlation was found in the gene expression level of TRF1 as well as TRF2 among different subtypes and stages of RCC as well as grades of clear cell RCC. Further, these findings were corroborated by immunohistochemical analysis, which revealed an increased expression of TRF1 and TRF2 proteins in different subtypes of RCC as well as also in different grades of clear cell RCC in comparison to normal renal parenchyma (Fig. 1B and 1C).

### TRF1 and TRF2 inhibition suppress proliferation and migration of RCC cells in vitro

**Fig 2:**
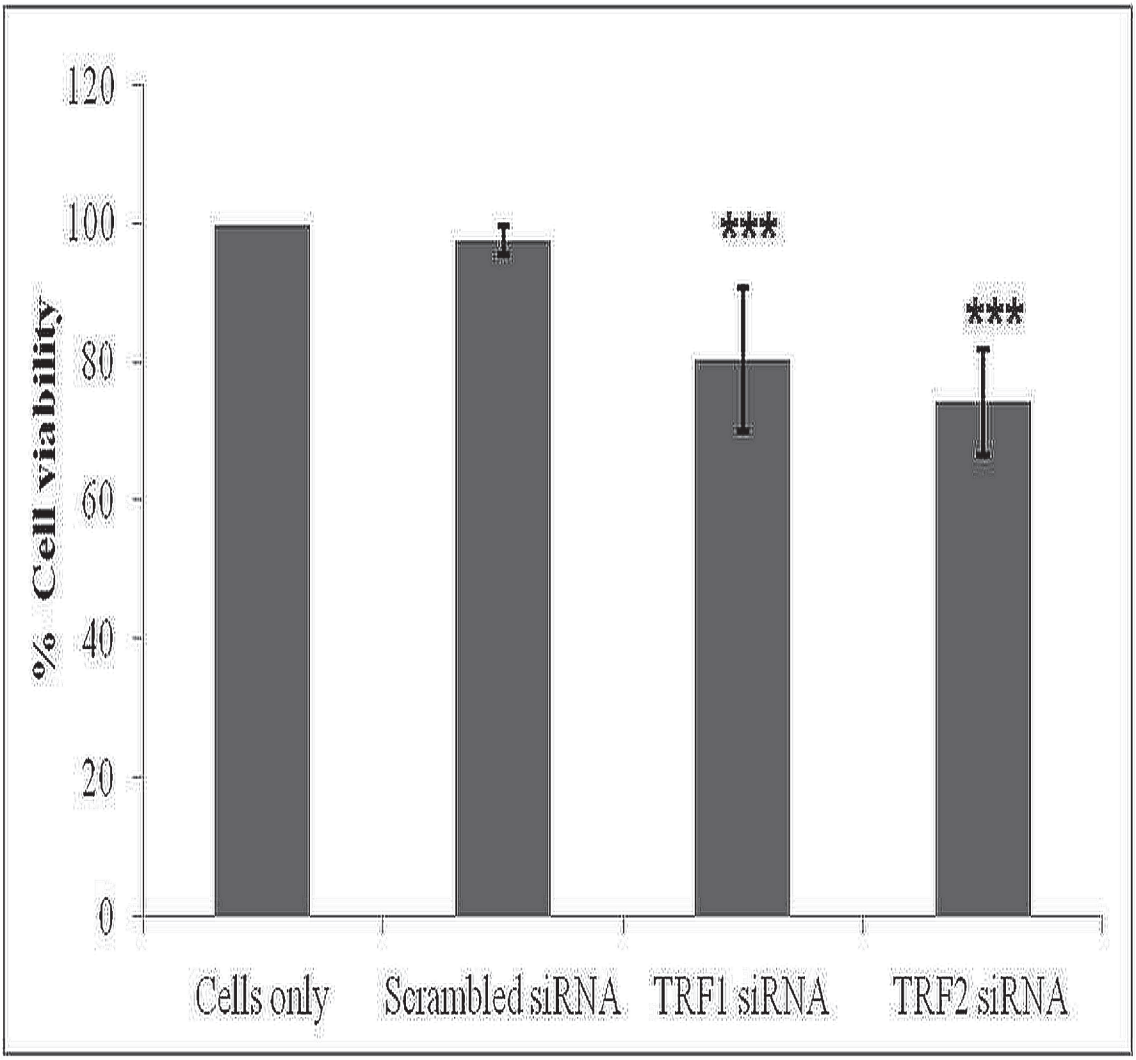
Antiproliferative effect of TRF1 or TRF2 silencing on RCC cells. Results were expressed as percentage of viable cells relative to untreated cells. Values are expressed as the percentage of control, which was defined as 100%. Each column represents the mean ± S.D. Results were analyzed by ANOVA. * P<0.05, ** P<0.01, *** P<0.001 was considered as significant.

**Fig 3:**
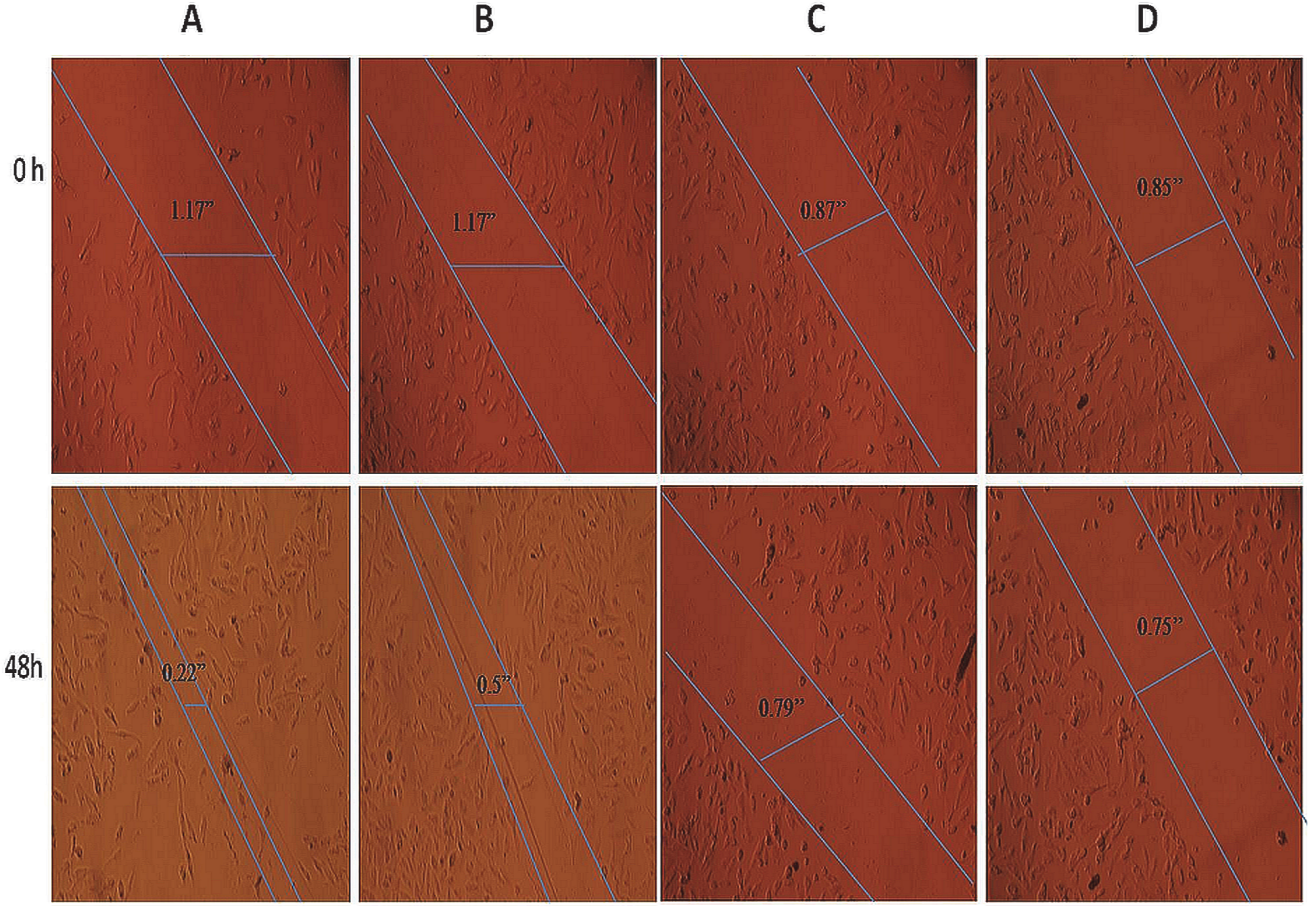
Inhibitory effect of TRF1, TRF2 silencing on the migration of renal cell carcinoma cells. Representative microscopic images are (A). Non-transfected cells (B). Cells transfected with scrambled siRNA (C). Cells transfected with TRF1 siRNA (D). Cells transfected with TRF2 siRNA. The solid lines define the area lacking cells.

Further, studies were carried out to investigate the role of TRF1 and TRF2 in cell proliferation and cell migration. In view of this fact, first the TRF1 and TRF2 gene expressions were knockdown using siRNA against them and it was observed by RT-PCR analysis that their gene expression was significantly reduced after respective siRNA treatment in RCC cells (A498) (S1 Fig.). Further, cell proliferation analysis using MTT assay in A498 cells revealed that percentage of viable cells was significantly reduced after siRNA mediated silencing of TRF1 or TRF2 expressions (P = 0.000; Fig. 2). In addition, wound healing assay showed significantly decreased migration of TRF1 or TRF2 siRNA treated cells as compared to untreated and scrambled siRNA treated cells (Fig. 3).

### TRF1 and TRF2 knockdown alter cell cycle progression and induce apoptosis

**Fig 4:**
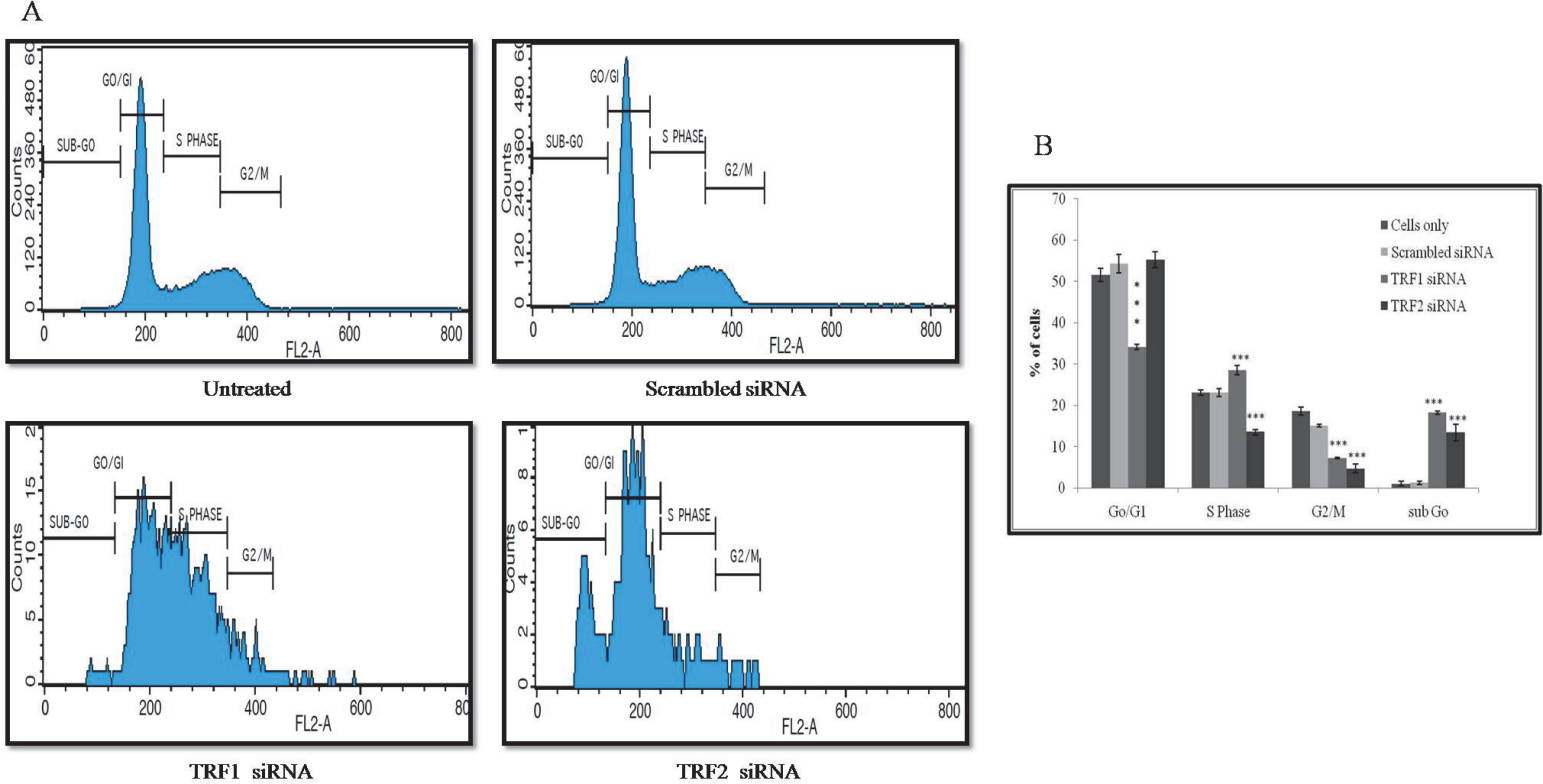
The effect of TRF1, TRF2 silencing on cell cycle distribution measured by flow cytometry (B) Graphical representation of flow cytrometry results. Values are expressed as the percentage of cells in each phase of cell cycle. Each column represents mean ± S.D. Results were analyzed by ANOVA. * P<0.05, ** P<0.01, *** P<0.001 was considered as significant.

The cell cycle distribution in A498 cells treated with scrambled siRNAs, TRF1 or TRF2 siRNAs for 96 hr are shown in Fig. 4. TRF1 inhibition results in alleviation in the percentage of cells in GO/G1 phase significantly (P = 0.000). Likewise, TRF2 inhibition significantly (P = 0.000) increased the percentage of cells in subG0 phase and G0/G1 phase as compared with untreated cells and scrambled siRNA treated cells. These results suggest that TRF1 and TRF2 inhibition arrest the cell cycle in S phase and G0/G1 phase respectively.

**Fig 5:**
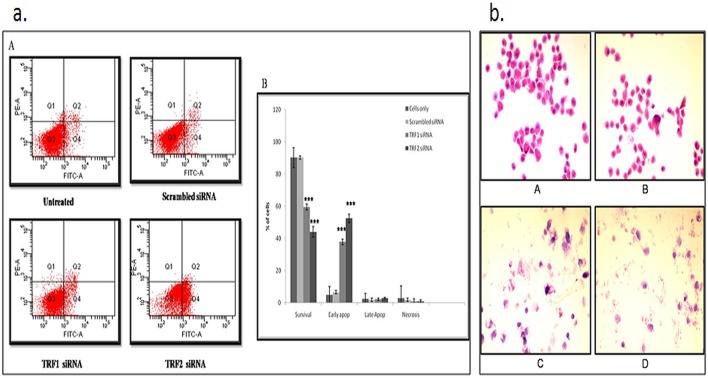
(a). Cytofluorimetric analysis of apoptosis by Annexin V-FITC/PI after TRF1, TRF2 silencing in renal cell carcinoma cells. (A) Viable cells are in the lower left quadrant, early apoptotic cells are in the lower right quadrant, late apoptotic or necrotic cells are in the upper right quadrant, and nonviable necrotic cells are in the upper left quadrant. (B) Graphical representation of flow cytometry results. Values are expressed as the percentage of cells in quadrant. Each column represents mean ± S.D. Results were analyzed by ANOVA. * P<0.05, ** P<0.01, *** P<0.001 was considered as significant. **(b). H& E staining of cells transfected with TRF1 or TRF2 siRNA.** (A) non-transfected cells (B) scrambled siRNA transfected cells (C) TRF1 siRNA transfected cells (D) TRF2 siRNA transfected cells.

Further, telomere dysfunction can induce DNA damage response pathway, such as apoptosis. Therefore, we examined whether TRF1, TRF2 gene silencing is associated with apoptosis. Flow cytometry analysis revealed that the morphology of cells was altered in the siRNA treated cell after 72 hr of treatment (S2 Fig.). Whereas, after 96 hr of treatment with siRNA cells showed apoptosis, in which 37% of TRF1 siRNA treated cells and 52.3% of TRF2 siRNA treated cells were in early apoptosis in comparison to untreated cells (4.2%) and scrambled siRNA treated cells (6.4%) (Fig. 5A). Moreover, we performed H & E staining of these cells, which showed the evidence of nuclear pyknosis, degeneration and cytoplasmic eosinophilia indicated apoptosis in the TRF1 or TRF2 siRNA treated cells as compared with untreated or scrambled siRNA treated cells (Fig. 5B). This observation further corroborated apoptosis induction in the cells after TRF1or TRF2 knockdown.

## Discussion

Telomeres are indispensible for the maintenance of genomic integrity. TRF1 and TRF2 bind to the telomere at double stranded region and entangle in the T-loop structure [19]. In the present study, up-regulation of TRF1 and TRF2 mRNA levels were observed in the RCC. Similarly, TRF1 and TRF2 protein expressions were also augmented in RCC tissues. These findings are in accordance with the previous reports where TRF1 and TRF2 expressions at both mRNA and protein levels were increased in other cancers such as human multistep carcinogenesis [20], gastric carcinoma [21] and lung carcinoma [12]. Taken together, these findings clearly suggest that TRF1 and TRF2 up-regulation could be a general mechanism in RCC.

It is noteworthy here that oxidative stress status has been implicated in the RCC pathogenesis [22]. Opresko *et al*. reported that mild oxidative stress can lead to augmented loss of telomeric DNA in human fibroblasts which can result in alteration of binding activity of TRF1 and TRF2 proteins [23]. Therefore, it is possible that DNA damage may result in the loss of critical telomere maintenance proteins. Thus, observed increased expression of TRF1 and TRF2 could be a compensatory mechanism in response to telomeric DNA damage in the present study. Multiple roles of TRF1 and TRF2 in telomere structure and function, as well as observed up-regulation of TRF1 and TRF2 in RCC have encouraged us to use these as interesting targets for anti-telomere pharmacological interventions. Therefore, this study was performed using siRNA to silence TRF1 and TRF2 expressions in RCC cell line (A498). It was observed that the viability and proliferation of A498 cells treated with TRF1 or TRF2 siRNA were reduced as compared to untreated and scrambled siRNA treated cells. Further, it was found that the reduced viability and cell proliferation in TRF1 or TRF2 knockdown cells were due to increase in apoptosis, which was accompanied by arrest in the cell cycle progression. These observations certainly support the involvement of TRF1 and TRF2 in maintaining the telomere structure. Inhibition of TRF1 and TRF2 may result in genomic instability due to which cells can undergo apoptosis. Since, it has been reported that the suppression of apoptosis involves TRF2. The loss of TRF2 leads to p53 and ATM dependent apoptosis as well as G1/S arrest [24]. This study also supports our observation that documented the cell cycle arrest in G1/S phase and induction of apoptosis after TRF2 silencing in RCC cells.

In the present study, inhibition of TRF1 and TRF2 expression had a differential effect on the cell cycle progression. TRF1 inhibition arrests the cell cycle progression in S-phase while TRF2 inhibition arrests the cell cycle progression at G1/S phase. These findings are of immense interest to explore the role and mechanism of actions of TRF1 and TRF2 in cell cycle progression. These findings support the conception that over-expression of TRF1 and TRF2 play an important role in RCC tumorigenesis. TRF2 inhibition is also reported to alleviate tumorigenesis in colorectal carcinoma by inducing apoptosis [25]. Increased TRF2 is the most critical candidate in telomere protection. However, the exact role of TRF2 in cancer development is not clear so far. Recently, Biroccio *et al.*, [26] have shown that a high level of TRF2 in tumor cells diminished their ability to recruit and activate natural killer cells density during the early development of human colon cancer.

Taken together, all of these findings conclude that up-regulation of TRF1 and TRF2 mRNA and protein level occurs in RCC. The silencing of telomere integrity preserver TRF1, TRF2 was done by using their siRNA which profoundly affect the ability of cells to proliferate and ultimately led to apoptosis in *in-vitro*. Therefore, antitumor therapy using TRF1 and TRF2 siRNA could be an effective treatment strategy in RCC.

## Supporting Information

S1 Fig RT-PCR analysis of TRF1 or TRF2 mRNA expression after transiently transfection with siRNA. A-498 cells were harvested after 48 hr of transfection and RT-PCR was performed to check the effect of gene silencing. β-actin was used an internal control. Real time PCR and the amplified products were run on 2% agarose gel.
(TIF)

S2 Fig Flow cytometry analysis of TRF1 or TRF2 silenced cells after 72hr of transaction showing the change in morphology. (A) Control; A498 cells (B) Scrambled siRNA treated cells (C) TRF1 siRNA treated cells (D) TRF2 siRNA treated cells.
(TIF)
