# Supplementary figures and images for: Over-Expression of Telomere Binding Factors (TRF1 & TRF2) in Renal Cell Carcinoma and Their Inhibition by Using SiRNA Induce Apoptosis, Reduce Cell Proliferation and Migration Invitro
Source: PLoS One. 2015 Mar 2;10(3):e0115651. doi: 10.1371/journal.pone.0115651 (PMC4346586; doi:10.1371/journal.pone.0115651)

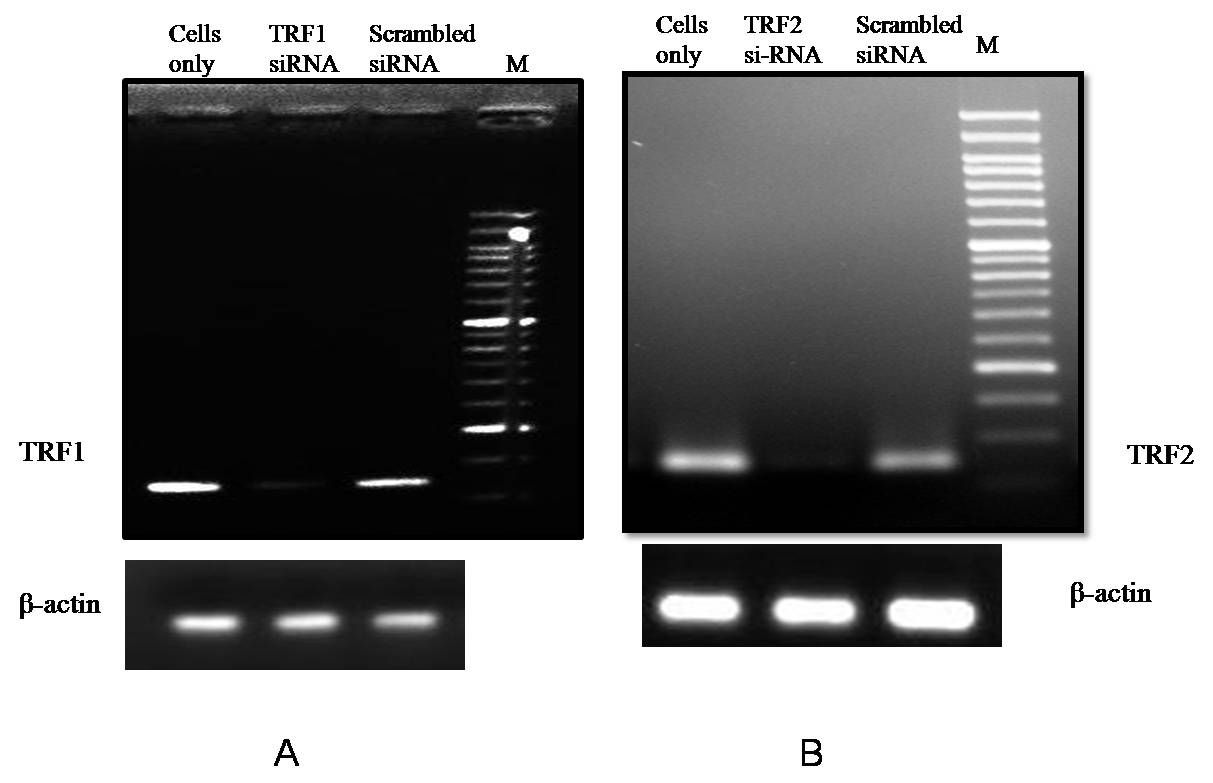

Supplement: S1 Fig — A-498 cells were harvested after 48 hr of transfection and RT-PCR was performed to check the effect of gene silencing. β-actin was used an internal control. Real time PCR and the amplified products were run on 2% agarose gel. (TIF) [file pone.0115651.s001.tif]

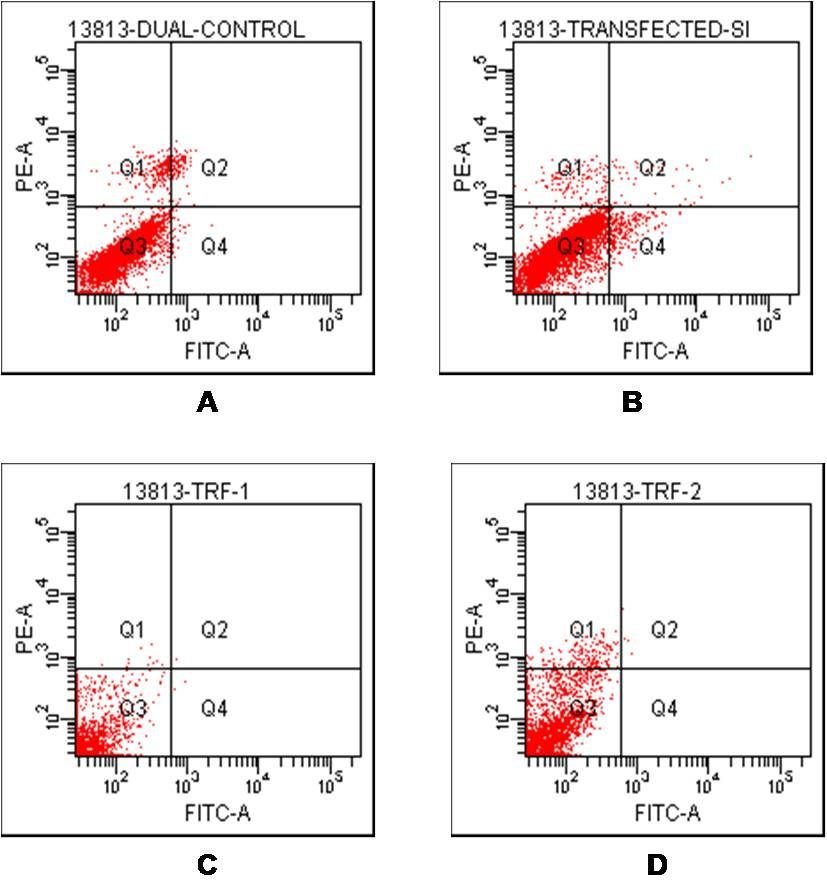

Supplement: S2 Fig — (A) Control; A498 cells (B) Scrambled siRNA treated cells (C) TRF1 siRNA treated cells (D) TRF2 siRNA treated cells. (TIF) [file pone.0115651.s002.tif]
